# The Retention of Vitamin D_2_ and 25-Hydroxyvitamin D_2_ in Pulse UV-Irradiated Dried Button Mushrooms (*Agaricus bisporus*) after 12 Months of Storage

**DOI:** 10.3390/foods12071429

**Published:** 2023-03-28

**Authors:** Glenn Cardwell, Janet F. Bornman, Anthony P. James, Alison Daly, Eleanor Dunlop, Georgios Dabos, Paul Adorno, Lucinda J. Black

**Affiliations:** 1 Curtin School of Population Health, Curtin University, Bentley, WA 6102, Australia; 2 Food Futures Institute, Murdoch University, Murdoch, WA 6150, Australia; 3 National Measurement Institute, Port Melbourne, VIC 3207, Australia

**Keywords:** *Agaricus bisporus*, 25-hydroxyvitamin D_2_, air-drying, mushroom, pulsed ultraviolet radiation, storage, vitamin D_2_

## Abstract

Fresh mushrooms exposed to ultraviolet (UV) radiation prior to drying generate high concentrations of vitamin D_2_. The aim of this study was to determine the retention of D vitamers in mushrooms that were pulse UV irradiated, then air dried, and stored for up to 12 months. Fresh button mushrooms (*A. bisporus*) were exposed to pulsed UV radiation (dose 200 mJ/cm^2^, peak of 17.5 W/cm^2^), air dried and vacuum sealed before being stored in the dark at room temperature. After storage, samples were freeze dried and quantified for D vitamers using triple quadrupole mass spectrometry. After 3, 6 and 12 months of storage, there was 100% (11.0 ± 0.8 µg/g dry weight (DW), 93% (10.1 ± 0.6 µg/g DW) and 58% (5.5 ± 0.6 µg/g DW) retention of vitamin D_2_ and 88% (0.14 ± 0.01 µg/g DW), 71% (0.11 ± 0.01 µg/g DW) and 68% (0.1 ± 0.01 µg/g DW) retention of 25-hydroxyvitamin D_2_ (25(OH)D_2_), respectively. Compared to the irradiated dried mushrooms that were not stored, the D vitamer concentration was statistically significantly lower (*p* < 0.05) at 6 and 12 months for 25(OH)D_2_ and at 12 months for vitamin D_2_. Sufficient vitamin D_2_ (99 µg) remained after 12 months storage to provide at least 100% of daily dietary vitamin D requirements in a 20 g serving.

## 1. Introduction

Vitamin D deficiency is prevalent worldwide, partly because there is low consumption of dietary vitamin D [1,2,3,4], and partly because of cultural norms or measures to reduce sun exposure to potentially damaging ultraviolet (UV) radiation [5,6]. Very few foods are a good source of vitamin D, the exceptions being meat, fish and egg yolk, and foods that have been fortified with vitamin D. Mushrooms generate vitamin D_2_ and 25-hydroxyvitamin D_2_ (25(OH)D_2_) when exposed to ultraviolet (UV) radiation such as solar radiation or UV lamps, with UV-B radiation being the most effective wavelength range [7,8,9]. UV radiation splits the B ring of ergosterol (pro-vitamin D_2_) between carbons 9 and 10, converting it into pre-vitamin D_2_. This structure is then rapidly rearranged in a heat-dependent process to an isomer known as ergocalciferol or vitamin D_2_ [10].

Cytochrome P450 enzymes present in the mushrooms appear to be responsible for hydroxylating vitamin D_2_ to 25(OH)D_2_ [11,12], although this occurs in mushrooms only if they are irradiated before drying and not vice versa [13]. Consumption of UV-irradiated mushrooms has been shown to increase serum 25(OH)D levels, particularly in consumers with a low vitamin D status (<50 nmol/L) [14,15]. There was little effect when the vitamin D status was high as the rise in 25(OH)D_2_ was offset by a fall in serum 25(OH)D_3_.

The amount of D vitamers produced by UV radiation will depend on the dose, the UV wavelength, the form of the mushrooms (sliced, whole, dried or powdered), the orientation of the mushrooms and the ambient temperature [7,16]. UV-exposed dried mushrooms can be a convenient and valuable source of vitamin D, especially for those wishing to limit their exposure to sunlight or avoid animal-derived dietary products [8,17,18].

Wild mushrooms that have been exposed to sunlight while growing or drying will naturally generate vitamin D_2_ [19,20,21]. Commercial dried mushrooms are normally sun-dried or hot-air dried, the former resulting in production of vitamin D_2_ [21,22]. Commercial dried mushrooms are often labelled with a shelf life of 12–18 months after packaging. Some countries permit dried mushrooms to be sold for up to 24 months after packaging, although there may be significant deterioration in quality and antioxidant activity beyond 12 months of storage [23].

Very little is known about the long-term retention of D vitamers in dried mushrooms, although there are some studies on both the short-term retention of vitamin D_2_ in fresh mushrooms and the long-term retention in frozen, powdered or dried mushrooms. The retention of vitamin D_2_ in UV-irradiated fresh mushrooms when refrigerated over periods of up to 14 days have showed varying results, with losses up to 50% [24,25,26,27]. Wild mushrooms retained virtually all their vitamin D_2_ after being frozen for 9 months [28]. Powdered, then pulse UV-irradiated oyster mushrooms stored for 60 days at room temperature (25 °C) or refrigerated (4 °C) retained 62% and 71% of their vitamin D_2_, respectively [29]. UV-B-irradiated, air-dried, button mushrooms stored at room temperature (22 °C) retained 54% of their vitamin D_2_ after 12 months and 48% after 18 months [17].

The long-term retention of both vitamin D_2_ and 25(OH)D_2_ has not yet been investigated in pulse UV-irradiated whole fresh button mushrooms (*Agaricus bisporus*) that were subsequently air dried. The aim of this study was to determine and compare the retention of vitamin D_2_ and 25(OH)D_2_ in button mushrooms that were pulse UV irradiated then hot-air dried, and packed and stored in similar conditions as retail dried mushrooms kept in the home.

## 2. Materials and Methods

### 2.1. Mushroom Samples

**Figure 1:**
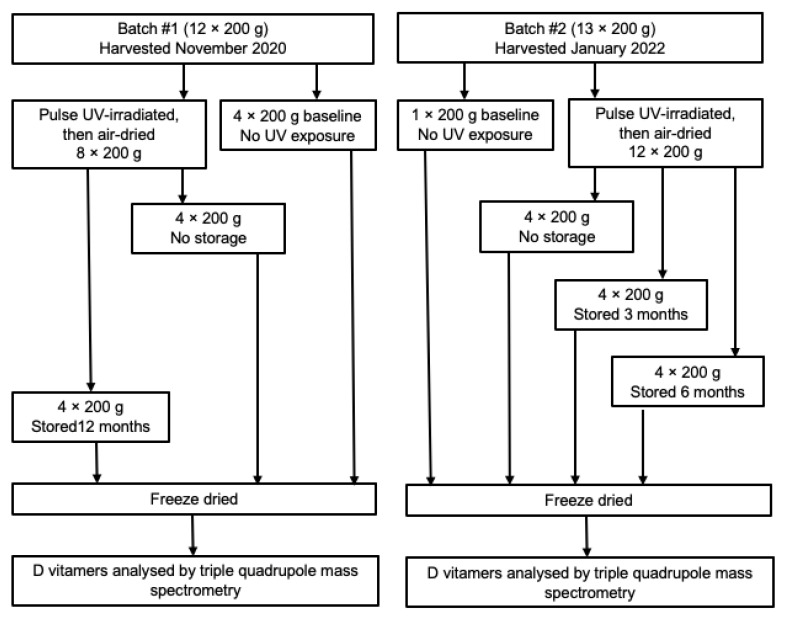
Schematic overview of UV-irradiating, drying and extracting D vitamers from button mushrooms.

In November 2020, twelve 200 g samples (Batch 1) of fresh button mushrooms (*Agaricus bisporus*), 4.5–5.5 cm in diameter and a mean weight of 28.6 g, were provided by a commercial farm (Costa Mushrooms) in Perth, Western Australia (Figure 1). A further thirteen 200 g samples of the same size (Batch 2) were collected from the same farm in January 2022. The mushrooms were harvested as they would be for market, with the stipe connected to the cap and the skirt intact.

The mushrooms were packed in boxes that were cooled, insulated and sealed from light, then transported by road to the laboratory at Curtin University (travel time 30 min). All samples were stored at 4 °C and processed within 48 h of collection. For 2 h prior to processing the samples were left at 23 °C, with any residual compost brushed off.

### 2.2. Baseline Samples

Baseline samples were analysed to confirm no unintended UV-radiation exposure, such as sunlight, had occurred prior to treatment. Four 200 g fresh samples were randomly selected from Batch 1 and one 200 g sample was randomly selected from Batch 2. The samples were sliced and placed in a freezer at −20 °C and lyophilised in a freeze dryer (Christ Alpha 1-2 LD plus, Martin Christ Gefriertrocknungsanlagen GmbH, Osterode am Harz, Germany) for 48 h at −30 °C and 37 pascals. The dry matter was made into a fine powder and stored at −20 °C.

### 2.3. UV-Irradiated then Air-Dried Samples (No Storage)

Four samples of 200 g whole mushrooms from both Batch 1 and Batch 2 were UV irradiated using a pulsed xenon lamp (Wek-tec XematicA-2L, Wek-tec e. K., Gottmadingen, Germany; emitting at 260–800 nm) with the skirted underside of each mushroom facing the lamp. The distance between the lamp and the mushrooms was 10 cm. The average dose of 200 mJ/cm^2^ and a peak of 17.5 W/cm^2^ were recorded with a radiometer (ILT800 CureRight, International Light Technologies, Peabody, MA, USA; measuring 215–350 nm).

After irradiation, the mushroom samples were placed in a convection oven and air dried for 18 h at 60 °C (Contherm Thermotec 2000, Contherm Scientific Ltd., Wellington, New Zealand). They were frozen at −20 °C and lyophilised for 24 h to remove any residual moisture and prepared for analysis as in Section 2.2. The shorter time for freeze-drying was due to these air-dried samples having a much lower moisture content compared to the fresh samples described in Section 2.2.

### 2.4. Storage for 3 and 6 Months

Eight 200 g samples from Batch 2 were UV irradiated and air dried as in Section 2.3. Each sample was individually vacuum sealed in plastic pouches (Laica Advanced Technology, Barbarano Mossano, Italy) and stored for either 3 or 6 months in a light-proof cardboard box at room temperature (January–July 2022). Storing in vacuum-sealed pouches was to mimic the processing of vacuum-packed commercial dried mushrooms that are then stored in a pantry by the consumer without any further exposure to UV radiation (household lights emit mainly visible light and very little UV radiation). During the first three months of storage the average temperature was 23 °C (range 22–25 °C) with an average humidity of 57% (range 43–65%) as measured by a temperature and humidity data logger (Lascar Electronics, EL-USB-2, Hong Kong, China). During the second three months of storage the average temperature was 21 °C (range 18–25 °C) with an average humidity of 53% (range 40–62%). After 3 and 6 months, samples were lyophilised for 24 h and prepared for analysis as in Section 2.2.

### 2.5. Storage for 12 Months

Four 200 g samples from Batch 1 were prepared as described in Section 2.3 and stored as in Section 2.4. The room temperature (av. 22.6 °C, range 20–26 °C; av. 50.7% humidity, range 47–62%) was measured during the 12 months storage (November 2020–November 2021). After 12 months, samples were lyophilised for 24 h and prepared for analysis as in Section 2.2.

### 2.6. Transport to Laboratory

After the designated storage time, samples were frozen at −20 °C prior to being sent by overnight courier in light-sealed, cooled, insulated containers for analysis at the National Measurement Institute (NMI) in Melbourne, Australia. On arrival, they were stored at −20 °C until analysis for vitamin D_2_ and 25(OH)D_2_, which occurred within 8 weeks.

### 2.7. Analysis of D-Vitamers

Analysis of each sample was by liquid chromatography coupled with triple quadrupole (LC-QQQ), tandem mass spectrometry. The analysis of D vitamers used NMI’s validated in-house method developed from published methodologies and approved by the National Association of Testing Authorities, Australia (ISO17025:2017) [30]. All samples were analysed in duplicate with the average of the two results being used. The limit of quantitation (LOQ) was 0.001 µg/g DW. The full method and verification has been previously reported [13].

### 2.8. Statistical Analysis

The Shapiro–Wilk test of normality was conducted on the storage times and retention of D vitamers. Wilcoxin Rank Sum tests were used to test associations between D vitamers at different storage times. Data were analysed using Stata (Version 17.0, StataCorp, College Station, TX, USA).

## 3. Results

The baseline vitamin D_2_ and 25(OH)D_2_ concentrations were both less than the LOQ, giving assurance there was no inadvertent UV radiation exposure prior to treatment. Table 1 shows the concentration of vitamin D_2_ and 25(OH)D_2_ in samples that were not stored, and after 3, 6 and 12 months of storage.

### 3.1. Vitamin D_2_

The average retention of vitamin D_2_ was 100% after three months of storage, and 93% after six months, neither being statistically significant different to the concentration in the samples that were not stored. However, only 58% of the original concentration was retained (*p* = 0.025) after 12 months storage.

### 3.2. 25-Hydroxyvitamin D_2_

The retention of 25(OH)D_2_ was 88% after three months which was not significantly different compared to the samples that were not stored. After 6 and 12 months of storage, there was a further drop to 71% and 68% retention, respectively, which was statistically significantly different to the original concentration (*p* = 0.029).

## 4. Discussion

### 4.1. Vitamin D_2_

There was little to no loss of vitamin D_2_ in whole UV-irradiated dried mushrooms stored for 3–6 months, with most of the loss occurring in the second six months of storage. There are only two studies on the long-term retention of vitamin D_2_ in UV-irradiated dried mushrooms. In a study, *A. bisporus* mushrooms were first irradiated, then air dried in a convection oven, before being stored in sealed bags at a room temperature of 20 ± 2 °C [17]. After 3, 8, 12 and 18 months, respectively, 81%, 65%, 54% and 48% of the original concentration of vitamin D_2_ remained. The retention of vitamin D_2_ after 12 months of storage was similar to our findings (25(OH)D_2_ was not measured). The second study was of oyster mushrooms that were freeze dried, powdered, then pulse UV irradiated and kept at room temperature (25 °C) without light exposure for 60 days. The vitamin D_2_ concentration dropped from the initial 65.4 to 40.8 µg/g DW at day 60, approximating a 62% retention of the original concentration [29].

Few studies have assessed the vitamin D_2_ retention in fresh UV-irradiated button mushrooms when stored at room temperature. Two studies found no evidence of vitamin D_2_ degradation, neither in whole mushrooms stored for 8 days at 25 °C [31], nor sliced mushrooms stored for 5 days at 30 °C [24]. In studies of the retention of vitamin D_2_ in UV-irradiated mushrooms during refrigeration the findings are not consistent. Vitamin D_2_ in UV-irradiated whole button mushrooms decreased by about half (from 14.8 to 6.2 µg/g DW) over 7 days at 4 °C [25]. In a study of pulse UV-irradiated sliced button mushrooms stored at 3 °C, the vitamin D_2_ dropped from 11.9 to 9.1 µg/g DW over three days then remained stable for a further 8 days [26]. Sliced button mushrooms refrigerated at 4 °C for four days showed no overall change in vitamin D_2_, although there was a rise and a fall in concentrations within that four days [24]. The vitamin D_2_ concentration was stable in white and brown whole button mushrooms, with a trend for the vitamin D_2_ to rise in the stems of brown button mushrooms, when stored at 4 °C for up to 14 d [27].

There appears to be good vitamin D_2_ retention in UV-irradiated dried mushrooms. The decline in vitamin D_2_ concentration in dried mushrooms occurs primarily after six months storage at 20–23 °C. However, if the original vitamin D_2_ concentration is sufficiently elevated (about 10 µg/g DW in this study and 14 µg/g DW in Sławińska et al. [17]), even if only half remains after 12 months storage there will still be 100–140 µg vitamin D_2_ in a 20 g serving, sufficient to satisfy vitamin D dietary requirements.

### 4.2. 25-Hydroxyvitamin D_2_

Fresh mushrooms were irradiated before air-drying as we have previously shown that irradiated mushrooms will generate 25(OH)D_2_, while irradiating dried mushrooms does not generate any 25(OH)D_2_ [13]. Another study has measured the concentration of 25(OH)D_2_ in button mushrooms with the concentration being below the level of detection [19]. Even at 0.1 µg 25(OH)D_2_/g DW, as found in the current study after 12 months storage, a 20 g serving will provide about 2.0 µg which is potentially nutritionally useful.

### 4.3. Effect of UV-Irradiation and Air-Drying

The concentration of D vitamers is likely to have been higher if the mushrooms were sliced or the gills were uncovered before UV exposure. Sliced mushrooms have a greater surface area for potential irradiation than whole mushrooms [26]. The gills of shiitake mushrooms have a higher concentration of ergosterol than the rest of the mushroom [32], while irradiating the gill side of button mushrooms produced more vitamin D_2_ than when the pileus side faced the UV-B radiation source [33].

After air-drying irradiated mushrooms, the low moisture content of the dried mushrooms may slow the rate of degradation of vitamin D_2_ [32]. The half-life of D_2_ in air-dried, UV-irradiated and powdered oyster mushrooms (*Pleurotus ostreatus*) was dependent on the storage temperature and water activity (a_w_) in the powder [34]. At a storage temperature of 20 °C and a water activity of 0.32 (a_w_ 0.32; distilled water has a a_w_ of 1.0), the half-life of vitamin D_2_ was 160 days, and 225 days in samples with a very low water activity (a_w_ 0.11).

### 4.4. Uses of Dried Vitamin D-Enriched Mushrooms

The UV dosage was chosen as it generated enough D vitamers to be of value in human nutrition. The serving size of dried mushrooms stated on commercial packs ranges between 10 and 30 g. Therefore, assuming a serving size of 20 g, our data show that a serving of irradiated, then air-dried mushrooms would offer approximately 99 µg D_2_ after 12 months storage. This is more than the vitamin D recommended daily intake in Australia (5–15 µg in adults) [35], Europe (15 µg) [36], the Nordic countries (10–20 µg) [37] and the USA (15–20 µg) [38]. The dose of UV radiation could be adjusted to generate D vitamer concentrations to suit local needs.

A higher radiation dose than used in this study would have generated greater amounts of the D vitamers, at a concentration potentially suitable for use as a powdered supplement. UV-irradiated whole, sliced or powdered dried mushrooms can be used as a supplemental vitamin D food product or an ingredient in functional foods, suited to those at a high risk of vitamin D deficiency [39]. The European Food Safety Authority has stated that UV-irradiated powdered mushrooms can be used as a novel food ingredient providing supplemental vitamin D_2_ [40]. Although the intended shelf life of the mushroom powder is two years, the results of their tests showed that the powder was stable for at least three years. The European Commission has also authorized the use of UV-treated mushrooms as a novel food [41].

Knowing the retention of D vitamers in stored dried mushrooms is useful when recommending a specific shelf-life and storage conditions. Although the long-term effect on the retention of vitamin D_2_ in UV-irradiated dried mushrooms, in particular button mushrooms, has not been well studied, the evidence from the current study suggests that sufficient vitamin D_2_ is present after 12 months of storage in darkness at 20–25 °C to be of nutritional significance.

The use of a validated analytical method that has been verified for a mushroom matrix was major strength of this study. In addition, there was a chain of custody from the farm to the laboratory, ensuring no unintended exposure to UV radiation. This was confirmed by analysing a fresh sample at the laboratory to ensure the concentrations of D-vitamers were below the LOQ. Although two different batches of mushrooms were analysed, they originated from the same farm under the same growing conditions, were irradiated and air dried with the same equipment and the same method and were analysed using the same method. The D vitamer concentrations measured prior to storage were similar in both batches. While pulsed UV lamps and machinery can initially be costly, the advantage of a pulsed radiation system is that the dose is consistent and can be varied depending on the concentration of D vitamers required. The D vitamers are generated in less than a second, while conventional UV lamps can take 30 min or more to generate a similar concentration. Therefore, the economics of commercially produced vitamin D-enriched mushrooms may favour the rapid throughput of pulse UV lamps.

## 5. Conclusions

Mushrooms are dried to increase the shelf life and make them easier to transport and store without refrigeration. UV-irradiated dried mushrooms are a good source of D vitamers. Vitamin D_2_ and 25(OH)D_2_ concentrations showed a gradual loss over 12 months, with the greatest rate of loss occurring during the latter 6 months. However, vacuum-sealed, UV-irradiated dried mushrooms kept at room temperature have a high retention of D-vitamers over 6 months after processing and packaging, a time span in which it is expected that most dried mushrooms would be cooked and consumed. Even after 12 months storage, UV-irradiated dried mushrooms still offered a valuable nutritional source of vitamin D_2_ in a 20 g serving size. As vitamin D deficiency is prevalent throughout the world, UV-irradiated dried mushrooms provide an opportunity to provide a convenient, long shelf-life source of vitamin D.

## Figures and Tables

**Table 1:** Retention of D vitamers in stored UV-irradiated, air-dried mushrooms.

	Fresh Weight, g (Mean ± SD)	Weight after Air-Drying, g	% Loss ^1^ (Mean ± SD)	Weight after Freeze-Drying, g	% Loss ^2^ (Mean ± SD)	D_2_ µg/g DW (Mean ± SD)	25(OH)D_2_ µg/g DW (Mean ± SD)
Mushrooms Batch #1							
Pulse irradiated, air dried, no storage (*n* = 4 × 200 g)	201.9 ± 2.4	19.7	90.2 ± 0.2	17.7	91.2 ± 0.2	9.5 ± 1.4 ^a^	0.14 ± 0.03 ^a^
Pulse irradiated, air dried, stored 12 months (*n* = 4 × 200 g)	214.4 ± 6.4	21.3	90.4 ± 0.7	19.2	91.1 ± 0.4	5.5 ± 0.6 ^a^ (57.7% retained)	0.10 ± 0.01 ^a^ (67.9% retained)
Mushrooms Batch #2							
Pulse irradiated, air dried, no storage (*n* = 4 × 200 g)	213.3 ± 3.6	21.6	89.9 ± 0.2	19.8	90.8 ± 0.2	10.9 ± 0.4	0.16 ± 0.02 ^b^
Pulse irradiated, air dried, stored 3 months (*n* = 4 × 200 g)	219.1 ± 11.1	21.0	90.4 ± 0.7	19.8	91.0 ± 0.2	11.0 ± 0.8 (100.1% retained)	0.14 ± 0.01 (88.1% retained)
Pulse irradiated, air dried, stored 6 months (*n* = 4 × 200 g)	217.6 ± 7.2	21.3	90.2 ± 1.2	20.0	90.8 ± 0.4	10.1 ± 0.6 (92.7% retained)	0.11 ± 0.01 ^b^ (71.1% retained)

25(OH)D_2_, 25-hydroxyvitamin D_2_; DW, dry weight; LOQ, level of quantitation; SD, standard deviation. LOQ < 0.001 µg/g. ^1^ % weight loss in the air-dried samples compared to the fresh weight. ^2^ % weight loss in the freeze-dried samples compared to the fresh weight. ^a^ Statistically significant difference (*p* < 0.05) between 0 and 12 months of storage. ^b^ Statistically significant difference (*p* < 0.05) between 0 and 6 months of storage.

## Data Availability

The data presented in this study are available upon request from the corresponding author. The data are not publicly available.
